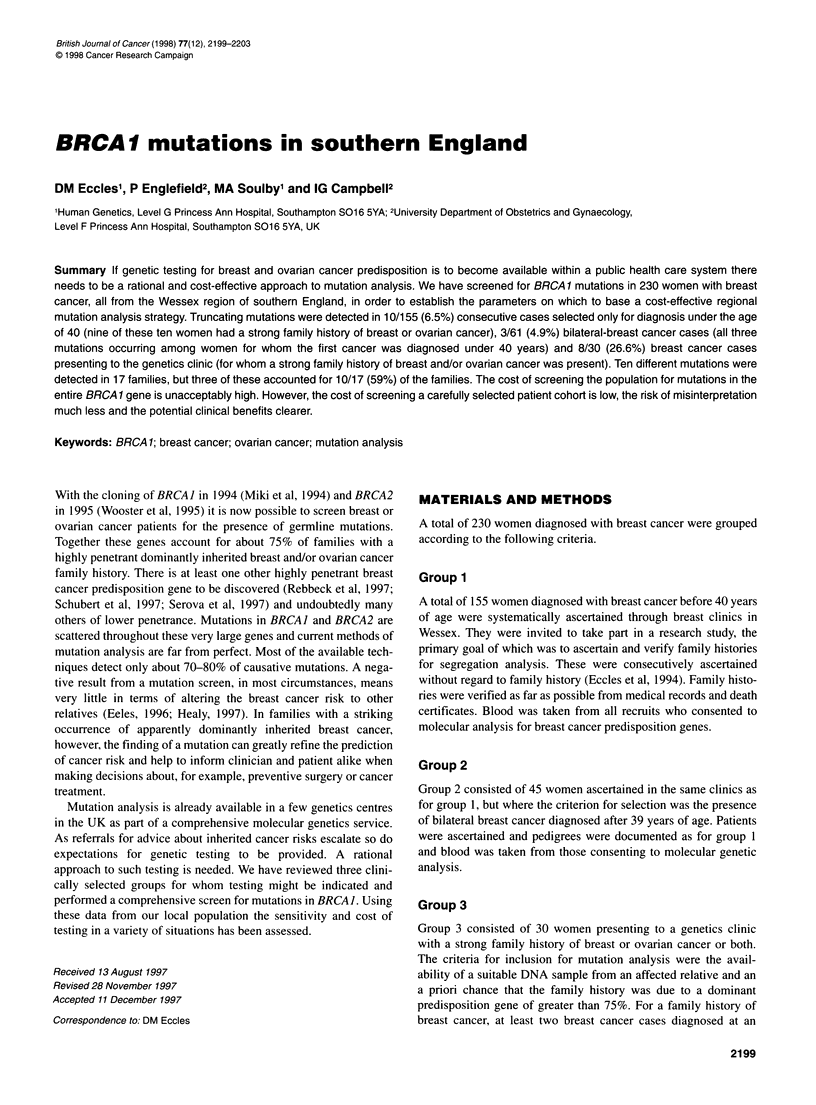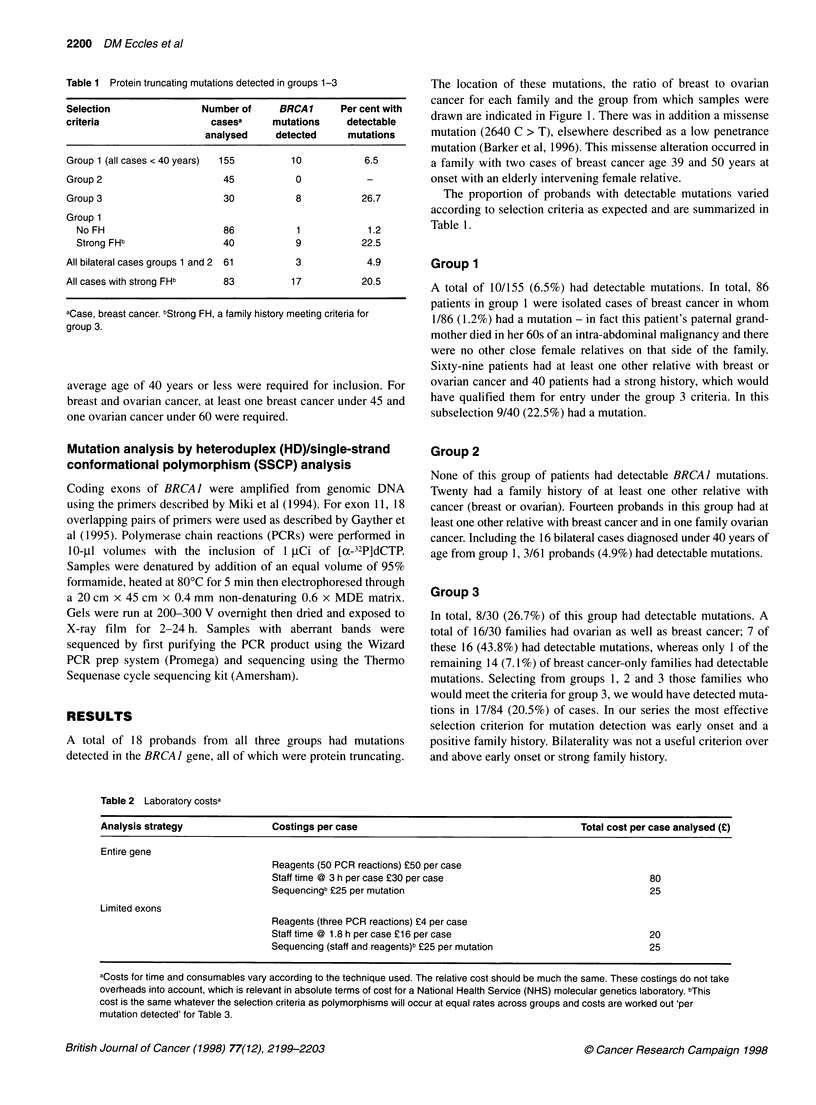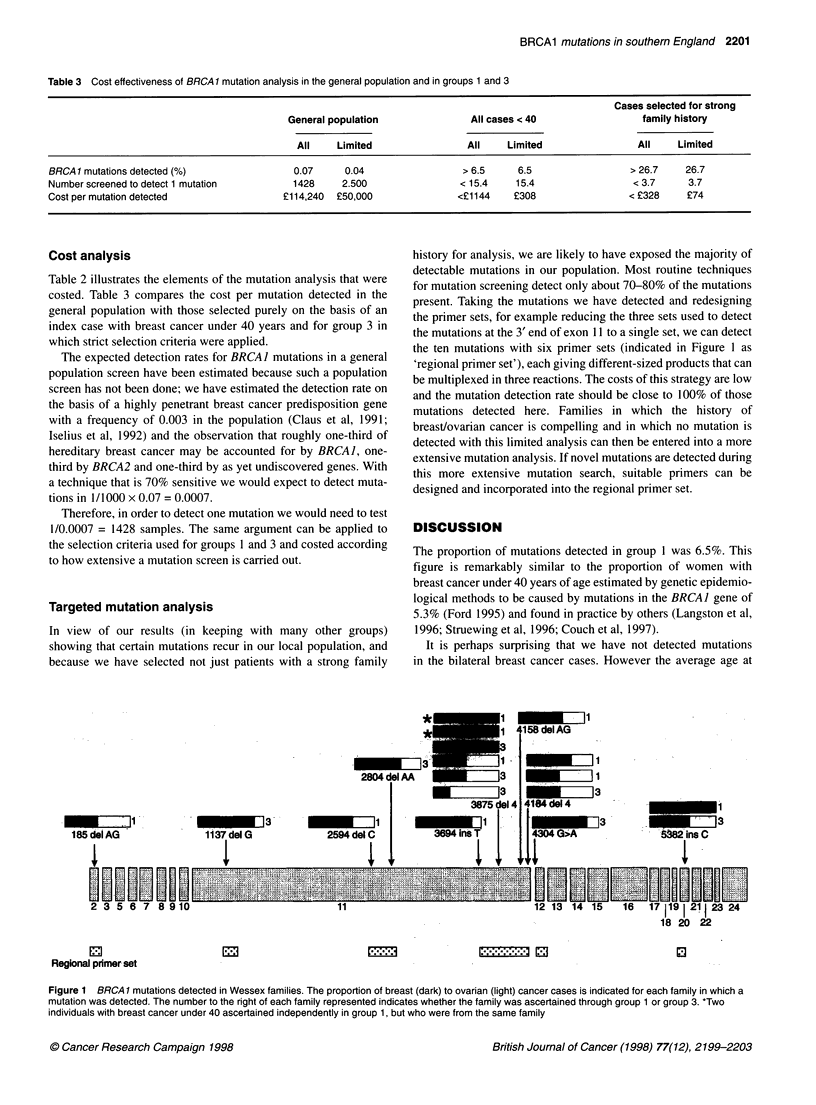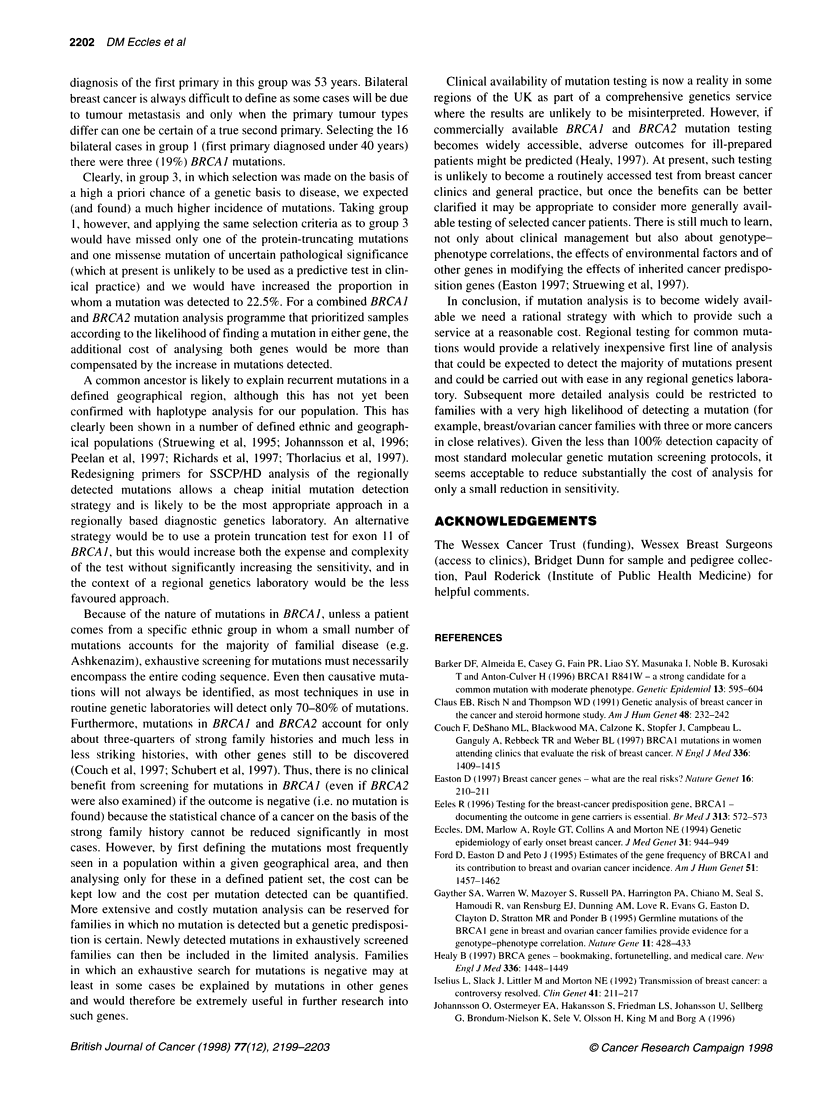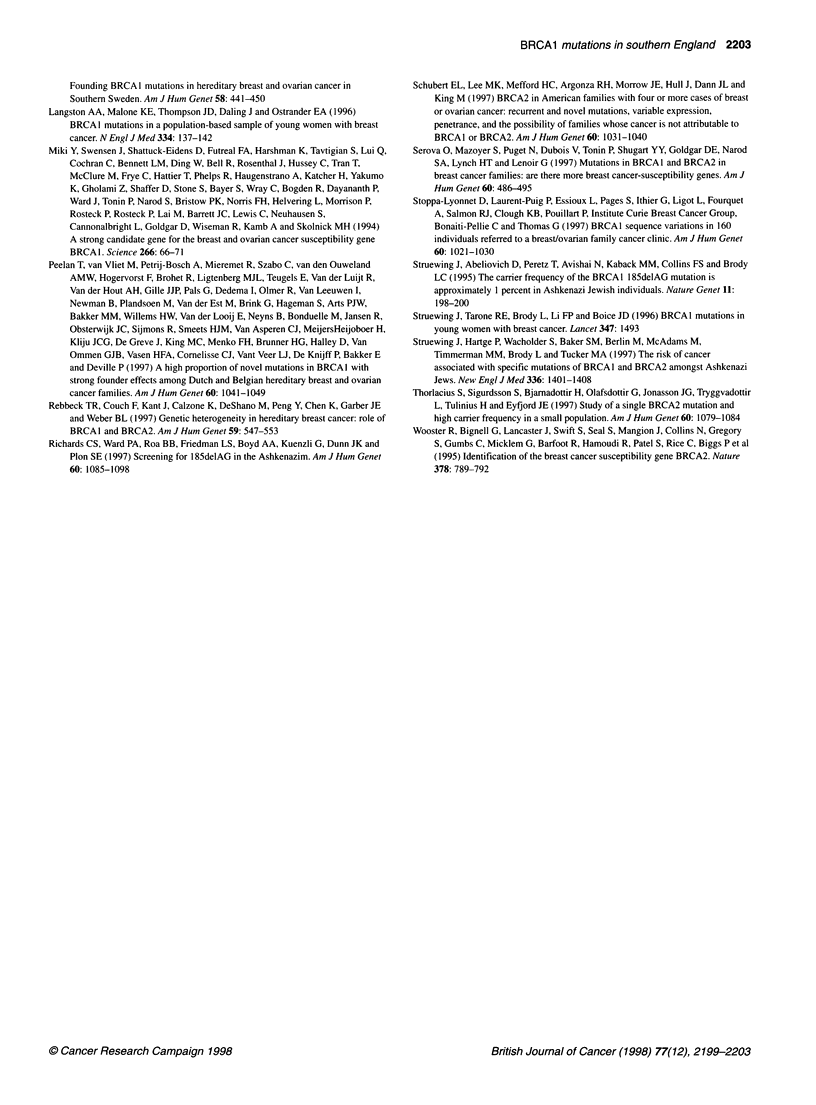# BRCA1 mutations in southern England.

**DOI:** 10.1038/bjc.1998.366

**Published:** 1998-06

**Authors:** D. M. Eccles, P. Englefield, M. A. Soulby, I. G. Campbell

**Affiliations:** Human Genetics, Level G Princess Ann Hospital, Southampton, UK.

## Abstract

If genetic testing for breast and ovarian cancer predisposition is to become available within a public health care system there needs to be a rational and cost-effective approach to mutation analysis. We have screened for BRCA1 mutations in 230 women with breast cancer, all from the Wessex region of southern England, in order to establish the parameters on which to base a cost-effective regional mutation analysis strategy. Truncating mutations were detected in 10/155 (6.5%) consecutive cases selected only for diagnosis under the age of 40 (nine of these ten women had a strong family history of breast or ovarian cancer), 3/61 (4.9%) bilateral-breast cancer cases (all three mutations occurring among women for whom the first cancer was diagnosed under 40 years) and 8/30 (26.6%) breast cancer cases presenting to the genetics clinic (for whom a strong family history of breast and/or ovarian cancer was present). Ten different mutations were detected in 17 families, but three of these accounted for 10/17 (59%) of the families. The cost of screening the population for mutations in the entire BRCA1 gene is unacceptably high. However, the cost of screening a carefully selected patient cohort is low, the risk of misinterpretation much less and the potential clinical benefits clearer.


British Journal of Cancer (1998) 77(12), 2199–2203
© 1998 Cancer Research Campaign

### *BRCA1* mutations in southern England

**DM Eccles[1], P Englefield[2], MA Soulby[1] and IG Campbell[2]**

[1]Human Genetics, Level G Princess Ann Hospital, Southampton SO16 5YA; [2]University Department of Obstetrics and Gynaecology, Level F Princess Ann Hospital, Southampton SO16 5YA, UK

**Summary** If genetic testing for breast and ovarian cancer predisposition is to become available within a public health care system there needs to be a rational and cost-effective approach to mutation analysis. We have screened for *BRCA1* mutations in 230 women with breast cancer, all from the Wessex region of southern England, in order to establish the parameters on which to base a cost-effective regional mutation analysis strategy. Truncating mutations were detected in 10/155 (6.5%) consecutive cases selected only for diagnosis under the age of 40 (nine of these ten women had a strong family history of breast or ovarian cancer), 3/61 (4.9%) bilateral-breast cancer cases (all three mutations occurring among women for whom the first cancer was diagnosed under 40 years) and 8/30 (26.6%) breast cancer cases presenting to the genetics clinic (for whom a strong family history of breast and/or ovarian cancer was present). Ten different mutations were detected in 17 families, but three of these accounted for 10/17 (59%) of the families. The cost of screening the population for mutations in the entire *BRCA1* gene is unacceptably high. However, the cost of screening a carefully selected patient cohort is low, the risk of misinterpretation much less and the potential clinical benefits clearer.

**Keywords:** *BRCA1*; breast cancer; ovarian cancer; mutation analysis

With the cloning of *BRCA1* in 1994 (Miki et al, 1994) and *BRCA2* in 1995 (Wooster et al, 1995) it is now possible to screen breast or ovarian cancer patients for the presence of germline mutations. Together these genes account for about 75% of families with a highly penetrant dominantly inherited breast and/or ovarian cancer family history. There is at least one other highly penetrant breast cancer predisposition gene to be discovered (Rebbeck et al, 1997; Schubert et al, 1997; Serova et al, 1997) and undoubtedly many others of lower penetrance. Mutations in *BRCA1* and *BRCA2* are scattered throughout these very large genes and current methods of mutation analysis are far from perfect. Most of the available techniques detect only about 70–80% of causative mutations. A negative result from a mutation screen, in most circumstances, means very little in terms of altering the breast cancer risk to other relatives (Eeles, 1996; Healy, 1997). In families with a striking occurrence of apparently dominantly inherited breast cancer, however, the finding of a mutation can greatly refine the prediction of cancer risk and help to inform clinician and patient alike when making decisions about, for example, preventive surgery or cancer treatment.

Mutation analysis is already available in a few genetics centres in the UK as part of a comprehensive molecular genetics service. As referrals for advice about inherited cancer risks escalate so do expectations for genetic testing to be provided. A rational approach to such testing is needed. We have reviewed three clinically selected groups for whom testing might be indicated and performed a comprehensive screen for mutations in *BRCA1*. Using these data from our local population the sensitivity and cost of testing in a variety of situations has been assessed.

*Received 13 August 1997*
*Revised 28 November 1997*
*Accepted 11 December 1997*

*Correspondence to:* DM Eccles

### MATERIALS AND METHODS

A total of 230 women diagnosed with breast cancer were grouped according to the following criteria.

### Group 1

A total of 155 women diagnosed with breast cancer before 40 years of age were systematically ascertained through breast clinics in Wessex. They were invited to take part in a research study, the primary goal of which was to ascertain and verify family histories for segregation analysis. These were consecutively ascertained without regard to family history (Eccles et al, 1994). Family histories were verified as far as possible from medical records and death certificates. Blood was taken from all recruits who consented to molecular analysis for breast cancer predisposition genes.

### Group 2

Group 2 consisted of 45 women ascertained in the same clinics as for group 1, but where the criterion for selection was the presence of bilateral breast cancer diagnosed after 39 years of age. Patients were ascertained and pedigrees were documented as for group 1 and blood was taken from those consenting to molecular genetic analysis.

### Group 3

Group 3 consisted of 30 women presenting to a genetics clinic with a strong family history of breast or ovarian cancer or both. The criteria for inclusion for mutation analysis were the availability of a suitable DNA sample from an affected relative and an a priori chance that the family history was due to a dominant predisposition gene of greater than 75%. For a family history of breast cancer, at least two breast cancer cases diagnosed at an

**Table 1** Protein truncating mutations detected in groups 1–3

| Selection criteria | Number of cases[a] analysed | *BRCA1* mutations detected | Per cent with detectable mutations |
|---|---|---|---|
| Group 1 (all cases < 40 years) | 155 | 10 | 6.5 |
| Group 2 | 45 | 0 | – |
| Group 3 | 30 | 8 | 26.7 |
| Group 1 | | | |
| No FH | 86 | 1 | 1.2 |
| Strong FH[b] | 40 | 9 | 22.5 |
| All bilateral cases groups 1 and 2 | 61 | 3 | 4.9 |
| All cases with strong FH[b] | 83 | 17 | 20.5 |

[a]Case, breast cancer. [b]Strong FH, a family history meeting criteria for group 3.

average age of 40 years or less were required for inclusion. For breast and ovarian cancer, at least one breast cancer under 45 and one ovarian cancer under 60 were required.

### Mutation analysis by heteroduplex (HD)/single-strand conformational polymorphism (SSCP) analysis

Coding exons of *BRCA1* were amplified from genomic DNA using the primers described by Miki et al (1994). For exon 11, 18 overlapping pairs of primers were used as described by Gayther et al (1995). Polymerase chain reactions (PCRs) were performed in 10-µl volumes with the inclusion of 1 µCi of [α-$^{32}$P]dCTP. Samples were denatured by addition of an equal volume of 95% formamide, heated at 80°C for 5 min then electrophoresed through a 20 cm × 45 cm × 0.4 mm non-denaturing 0.6 × MDE matrix. Gels were run at 200–300 V overnight then dried and exposed to X-ray film for 2–24 h. Samples with aberrant bands were sequenced by first purifying the PCR product using the Wizard PCR prep system (Promega) and sequencing using the Thermo Sequenase cycle sequencing kit (Amersham).

### RESULTS

A total of 18 probands from all three groups had mutations detected in the *BRCA1* gene, all of which were protein truncating. The location of these mutations, the ratio of breast to ovarian cancer for each family and the group from which samples were drawn are indicated in Figure 1. There was in addition a missense mutation (2640 C > T), elsewhere described as a low penetrance mutation (Barker et al, 1996). This missense alteration occurred in a family with two cases of breast cancer age 39 and 50 years at onset with an elderly intervening female relative.

The proportion of probands with detectable mutations varied according to selection criteria as expected and are summarized in Table 1.

### Group 1

A total of 10/155 (6.5%) had detectable mutations. In total, 86 patients in group 1 were isolated cases of breast cancer in whom 1/86 (1.2%) had a mutation – in fact this patient's paternal grandmother died in her 60s of an intra-abdominal malignancy and there were no other close female relatives on that side of the family. Sixty-nine patients had at least one other relative with breast or ovarian cancer and 40 patients had a strong history, which would have qualified them for entry under the group 3 criteria. In this subselection 9/40 (22.5%) had a mutation.

### Group 2

None of this group of patients had detectable *BRCA1* mutations. Twenty had a family history of at least one other relative with cancer (breast or ovarian). Fourteen probands in this group had at least one other relative with breast cancer and in one family ovarian cancer. Including the 16 bilateral cases diagnosed under 40 years of age from group 1, 3/61 probands (4.9%) had detectable mutations.

### Group 3

In total, 8/30 (26.7%) of this group had detectable mutations. A total of 16/30 families had ovarian as well as breast cancer; 7 of these 16 (43.8%) had detectable mutations, whereas only 1 of the remaining 14 (7.1%) of breast cancer-only families had detectable mutations. Selecting from groups 1, 2 and 3 those families who would meet the criteria for group 3, we would have detected mutations in 17/84 (20.5%) of cases. In our series the most effective selection criterion for mutation detection was early onset and a positive family history. Bilaterality was not a useful criterion over and above early onset or strong family history.

**Table 2** Laboratory costs[a]

| Analysis strategy | Costings per case | Total cost per case analysed (£) |
|---|---|---|
| Entire gene | | |
| | Reagents (50 PCR reactions) £50 per case | |
| | Staff time @ 3 h per case £30 per case | 80 |
| | Sequencing[b] £25 per mutation | 25 |
| Limited exons | | |
| | Reagents (three PCR reactions) £4 per case | |
| | Staff time @ 1.8 h per case £16 per case | 20 |
| | Sequencing (staff and reagents)[b] £25 per mutation | 25 |

[a]Costs for time and consumables vary according to the technique used. The relative cost should be much the same. These costings do not take overheads into account, which is relevant in absolute terms of cost for a National Health Service (NHS) molecular genetics laboratory. [b]This cost is the same whatever the selection criteria as polymorphisms will occur at equal rates across groups and costs are worked out 'per mutation detected' for Table 3.

 © Cancer Research Campaign 1998

**Table 3** Cost effectiveness of *BRCA1* mutation analysis in the general population and in groups 1 and 3

| | General population | | All cases < 40 | | Cases selected for strong family history | |
|---|---|---|---|---|---|---|
| | All | Limited | All | Limited | All | Limited |
| *BRCA1* mutations detected (%) | 0.07 | 0.04 | > 6.5 | 6.5 | > 26.7 | 26.7 |
| Number screened to detect 1 mutation | 1428 | 2.500 | < 15.4 | 15.4 | < 3.7 | 3.7 |
| Cost per mutation detected | £114,240 | £50,000 | <£1144 | £308 | < £328 | £74 |

### Cost analysis

Table 2 illustrates the elements of the mutation analysis that were costed. Table 3 compares the cost per mutation detected in the general population with those selected purely on the basis of an index case with breast cancer under 40 years and for group 3 in which strict selection criteria were applied.

The expected detection rates for *BRCA1* mutations in a general population screen have been estimated because such a population screen has not been done; we have estimated the detection rate on the basis of a highly penetrant breast cancer predisposition gene with a frequency of 0.003 in the population (Claus et al, 1991; Iselius et al, 1992) and the observation that roughly one-third of hereditary breast cancer may be accounted for by *BRCA1*, one-third by *BRCA2* and one-third by as yet undiscovered genes. With a technique that is 70% sensitive we would expect to detect mutations in $1/1000 \times 0.07 = 0.0007$.

Therefore, in order to detect one mutation we would need to test $1/0.0007 = 1428$ samples. The same argument can be applied to the selection criteria used for groups 1 and 3 and costed according to how extensive a mutation screen is carried out.

### Targeted mutation analysis

In view of our results (in keeping with many other groups) showing that certain mutations recur in our local population, and because we have selected not just patients with a strong family history for analysis, we are likely to have exposed the majority of detectable mutations in our population. Most routine techniques for mutation screening detect only about 70–80% of the mutations present. Taking the mutations we have detected and redesigning the primer sets, for example reducing the three sets used to detect the mutations at the 3′ end of exon 11 to a single set, we can detect the ten mutations with six primer sets (indicated in Figure 1 as 'regional primer set'), each giving different-sized products that can be multiplexed in three reactions. The costs of this strategy are low and the mutation detection rate should be close to 100% of those mutations detected here. Families in which the history of breast/ovarian cancer is compelling and in which no mutation is detected with this limited analysis can then be entered into a more extensive mutation analysis. If novel mutations are detected during this more extensive mutation search, suitable primers can be designed and incorporated into the regional primer set.

### DISCUSSION

The proportion of mutations detected in group 1 was 6.5%. This figure is remarkably similar to the proportion of women with breast cancer under 40 years of age estimated by genetic epidemiological methods to be caused by mutations in the *BRCA1* gene of 5.3% (Ford 1995) and found in practice by others (Langston et al, 1996; Struewing et al, 1996; Couch et al, 1997).

It is perhaps surprising that we have not detected mutations in the bilateral breast cancer cases. However the average age at

**Figure 1** *BRCA1* mutations detected in Wessex families. The proportion of breast (dark) to ovarian (light) cancer cases is indicated for each family in which a mutation was detected. The number to the right of each family represented indicates whether the family was ascertained through group 1 or group 3. *Two individuals with breast cancer under 40 ascertained independently in group 1, but who were from the same family

© Cancer Research Campaign 1998

diagnosis of the first primary in this group was 53 years. Bilateral breast cancer is always difficult to define as some cases will be due to tumour metastasis and only when the primary tumour types differ can one be certain of a true second primary. Selecting the 16 bilateral cases in group 1 (first primary diagnosed under 40 years) there were three (19%) *BRCA1* mutations.

Clearly, in group 3, in which selection was made on the basis of a high a priori chance of a genetic basis to disease, we expected (and found) a much higher incidence of mutations. Taking group 1, however, and applying the same selection criteria as to group 3 would have missed only one of the protein-truncating mutations and one missense mutation of uncertain pathological significance (which at present is unlikely to be used as a predictive test in clinical practice) and we would have increased the proportion in whom a mutation was detected to 22.5%. For a combined *BRCA1* and *BRCA2* mutation analysis programme that prioritized samples according to the likelihood of finding a mutation in either gene, the additional cost of analysing both genes would be more than compensated by the increase in mutations detected.

A common ancestor is likely to explain recurrent mutations in a defined geographical region, although this has not yet been confirmed with haplotype analysis for our population. This has clearly been shown in a number of defined ethnic and geographical populations (Struewing et al, 1995; Johannsson et al, 1996; Peelan et al, 1997; Richards et al, 1997; Thorlacius et al, 1997). Redesigning primers for SSCP/HD analysis of the regionally detected mutations allows a cheap initial mutation detection strategy and is likely to be the most appropriate approach in a regionally based diagnostic genetics laboratory. An alternative strategy would be to use a protein truncation test for exon 11 of *BRCA1*, but this would increase both the expense and complexity of the test without significantly increasing the sensitivity, and in the context of a regional genetics laboratory would be the less favoured approach.

Because of the nature of mutations in *BRCA1*, unless a patient comes from a specific ethnic group in whom a small number of mutations accounts for the majority of familial disease (e.g. Ashkenazim), exhaustive screening for mutations must necessarily encompass the entire coding sequence. Even then causative mutations will not always be identified, as most techniques in use in routine genetic laboratories will detect only 70–80% of mutations. Furthermore, mutations in *BRCA1* and *BRCA2* account for only about three-quarters of strong family histories and much less in less striking histories, with other genes still to be discovered (Couch et al, 1997; Schubert et al, 1997). Thus, there is no clinical benefit from screening for mutations in *BRCA1* (even if *BRCA2* were also examined) if the outcome is negative (i.e. no mutation is found) because the statistical chance of a cancer on the basis of the strong family history cannot be reduced significantly in most cases. However, by first defining the mutations most frequently seen in a population within a given geographical area, and then analysing only for these in a defined patient set, the cost can be kept low and the cost per mutation detected can be quantified. More extensive and costly mutation analysis can be reserved for families in which no mutation is detected but a genetic predisposition is certain. Newly detected mutations in exhaustively screened families can then be included in the limited analysis. Families in which an exhaustive search for mutations is negative may at least in some cases be explained by mutations in other genes and would therefore be extremely useful in further research into such genes.

Clinical availability of mutation testing is now a reality in some regions of the UK as part of a comprehensive genetics service where the results are unlikely to be misinterpreted. However, if commercially available *BRCA1* and *BRCA2* mutation testing becomes widely accessible, adverse outcomes for ill-prepared patients might be predicted (Healy, 1997). At present, such testing is unlikely to become a routinely accessed test from breast cancer clinics and general practice, but once the benefits can be better clarified it may be appropriate to consider more generally available testing of selected cancer patients. There is still much to learn, not only about clinical management but also about genotype–phenotype correlations, the effects of environmental factors and of other genes in modifying the effects of inherited cancer predisposition genes (Easton 1997; Struewing et al, 1997).

In conclusion, if mutation analysis is to become widely available we need a rational strategy with which to provide such a service at a reasonable cost. Regional testing for common mutations would provide a relatively inexpensive first line of analysis that could be expected to detect the majority of mutations present and could be carried out with ease in any regional genetics laboratory. Subsequent more detailed analysis could be restricted to families with a very high likelihood of detecting a mutation (for example, breast/ovarian cancer families with three or more cancers in close relatives). Given the less than 100% detection capacity of most standard molecular genetic mutation screening protocols, it seems acceptable to reduce substantially the cost of analysis for only a small reduction in sensitivity.

### ACKNOWLEDGEMENTS

The Wessex Cancer Trust (funding), Wessex Breast Surgeons (access to clinics), Bridget Dunn for sample and pedigree collection, Paul Roderick (Institute of Public Health Medicine) for helpful comments.

### REFERENCES

Barker DF, Almeida E, Casey G, Fain PR, Liao SY, Masunaka I, Noble B, Kurosaki T and Anton-Culver H (1996) BRCA1 R841W – a strong candidate for a common mutation with moderate phenotype. *Genetic Epidemiol* **13**: 595–604

Claus EB, Risch N and Thompson WD (1991) Genetic analysis of breast cancer in the cancer and steroid hormone study. *Am J Hum Genet* **48**: 232–242

Couch F, DeShano ML, Blackwood MA, Calzone K, Stopfer J, Campbeau L, Ganguly A, Rebbeck TR and Weber BL (1997) BRCA1 mutations in women attending clinics that evaluate the risk of breast cancer. *N Engl J Med* **336**: 1409–1415

Easton D (1997) Breast cancer genes – what are the real risks? *Nature Genet* **16**: 210–211

Eeles R (1996) Testing for the breast-cancer predisposition gene, BRCA1 – documenting the outcome in gene carriers is essential. *Br Med J* **313**: 572–573

Eccles, DM, Marlow A, Royle GT, Collins A and Morton NE (1994) Genetic epidemiology of early onset breast cancer. *J Med Genet* **31**: 944–949

Ford D, Easton D and Peto J (1995) Estimates of the gene frequency of BRCA1 and its contribution to breast and ovarian cancer incidence. *Am J Hum Genet* **51**: 1457–1462

Gayther SA, Warren W, Mazoyer S, Russell PA, Harrington PA, Chiano M, Seal S, Hamoudi R, van Rensburg EJ, Dunning AM, Love R, Evans G, Easton D, Clayton D, Stratton MR and Ponder B (1995) Germline mutations of the BRCA1 gene in breast and ovarian cancer families provide evidence for a genotype–phenotype correlation. *Nature Gene* **11**: 428–433

Healy B (1997) BRCA genes – bookmaking, fortunetelling, and medical care. *New Engl J Med* **336**: 1448–1449

Iselius L, Slack J, Littler M and Morton NE (1992) Transmission of breast cancer: a controversy resolved. *Clin Genet* **41**: 211–217

Johannsson O, Ostermeyer EA, Hakansson S, Friedman LS, Johansson U, Sellberg G, Brondum-Nielson K, Sele V, Olsson H, King M and Borg A (1996)

© Cancer Research Campaign 1998

Founding BRCA1 mutations in hereditary breast and ovarian cancer in Southern Sweden. *Am J Hum Genet* **58**: 441–450

Langston AA, Malone KE, Thompson JD, Daling J and Ostrander EA (1996) BRCA1 mutations in a population-based sample of young women with breast cancer. *N Engl J Med* **334**: 137–142

Miki Y, Swensen J, Shattuck-Eidens D, Futreal FA, Harshman K, Tavtigian S, Lui Q, Cochran C, Bennett LM, Ding W, Bell R, Rosenthal J, Hussey C, Tran T, McClure M, Frye C, Hattier T, Phelps R, Haugenstrano A, Katcher H, Yakumo K, Gholami Z, Shaffer D, Stone S, Bayer S, Wray C, Bogden R, Dayananth P, Ward J, Tonin P, Narod S, Bristow PK, Norris FH, Helvering L, Morrison P, Rosteck P, Rosteck P, Lai M, Barrett JC, Lewis C, Neuhausen S, Cannonalbright L, Goldgar D, Wiseman R, Kamb A and Skolnick MH (1994) A strong candidate gene for the breast and ovarian cancer susceptibility gene BRCA1. *Science* **266**: 66–71

Peelan T, van Vliet M, Petrij-Bosch A, Mieremet R, Szabo C, van den Ouweland AMW, Hogervorst F, Brohet R, Ligtenberg MJL, Teugels E, Van der Luijt R, Van der Hout AH, Gille JJP, Pals G, Dedema I, Olmer R, Van Leeuwen I, Newman B, Plandsoen M, Van der Est M, Brink G, Hageman S, Arts PJW, Bakker MM, Willems HW, Van der Looij E, Neyns B, Bonduelle M, Jansen R, Obsterwijk JC, Sijmons R, Smeets HJM, Van Asperen CJ, MeijersHeijoboer H, Kliju JCG, De Greve J, King MC, Menko FH, Brunner HG, Halley D, Van Ommen GJB, Vasen HFA, Cornelisse CJ, Vant Veer LJ, De Knijff P, Bakker E and Deville P (1997) A high proportion of novel mutations in BRCA1 with strong founder effects among Dutch and Belgian hereditary breast and ovarian cancer families. *Am J Hum Genet* **60**: 1041–1049

Rebbeck TR, Couch F, Kant J, Calzone K, DeShano M, Peng Y, Chen K, Garber JE and Weber BL (1997) Genetic heterogeneity in hereditary breast cancer: role of BRCA1 and BRCA2. *Am J Hum Genet* **59**: 547–553

Richards CS, Ward PA, Roa BB, Friedman LS, Boyd AA, Kuenzli G, Dunn JK and Plon SE (1997) Screening for 185delAG in the Ashkenazim. *Am J Hum Genet* **60**: 1085–1098

Schubert EL, Lee MK, Mefford HC, Argonza RH, Morrow JE, Hull J, Dann JL and King M (1997) BRCA2 in American families with four or more cases of breast or ovarian cancer: recurrent and novel mutations, variable expression, penetrance, and the possibility of families whose cancer is not attributable to BRCA1 or BRCA2. *Am J Hum Genet* **60**: 1031–1040

Serova O, Mazoyer S, Puget N, Dubois V, Tonin P, Shugart YY, Goldgar DE, Narod SA, Lynch HT and Lenoir G (1997) Mutations in BRCA1 and BRCA2 in breast cancer families: are there more breast cancer-susceptibility genes. *Am J Hum Genet* **60**: 486–495

Stoppa-Lyonnet D, Laurent-Puig P, Essioux L, Pages S, Ithier G, Ligot L, Fourquet A, Salmon RJ, Clough KB, Pouillart P, Institute Curie Breast Cancer Group, Bonaiti-Pellie C and Thomas G (1997) BRCA1 sequence variations in 160 individuals referred to a breast/ovarian family cancer clinic. *Am J Hum Genet* **60**: 1021–1030

Struewing J, Abeliovich D, Peretz T, Avishai N, Kaback MM, Collins FS and Brody LC (1995) The carrier frequency of the BRCA1 185delAG mutation is approximately 1 percent in Ashkenazi Jewish individuals. *Nature Genet* **11**: 198–200

Struewing J, Tarone RE, Brody L, Li FP and Boice JD (1996) BRCA1 mutations in young women with breast cancer. *Lancet* **347**: 1493

Struewing J, Hartge P, Wacholder S, Baker SM, Berlin M, McAdams M, Timmerman MM, Brody L and Tucker MA (1997) The risk of cancer associated with specific mutations of BRCA1 and BRCA2 amongst Ashkenazi Jews. *New Engl J Med* **336**: 1401–1408

Thorlacius S, Sigurdsson S, Bjarnadottir H, Olafsdottir G, Jonasson JG, Tryggvadottir L, Tulinius H and Eyfjord JE (1997) Study of a single BRCA2 mutation and high carrier frequency in a small population. *Am J Hum Genet* **60**: 1079–1084

Wooster R, Bignell G, Lancaster J, Swift S, Seal S, Mangion J, Collins N, Gregory S, Gumbs C, Micklem G, Barfoot R, Hamoudi R, Patel S, Rice C, Biggs P et al (1995) Identification of the breast cancer susceptibility gene BRCA2. *Nature* **378**: 789–792

© Cancer Research Campaign 1998